# Single crystal structures and theoretical calculations of uranium endohedral metallofullerenes (U@*C*_2*n*_, 2*n* = 74, 82) show cage isomer dependent oxidation states for U†
†Electronic supplementary information (ESI) available. CCDC 150850815085091522558. For ESI and crystallographic data in CIF or other electronic format see DOI: 10.1039/c7sc01711a


**DOI:** 10.1039/c7sc01711a

**Published:** 2017-05-22

**Authors:** Wenting Cai, Roser Morales-Martínez, Xingxing Zhang, Daniel Najera, Elkin L. Romero, Alejandro Metta-Magaña, Antonio Rodríguez-Fortea, Skye Fortier, Ning Chen, Josep M. Poblet, Luis Echegoyen

**Affiliations:** a Department of Chemistry , University of Texas at El Paso , 500 W University Avenue , El Paso , Texas 79968 , USA . Email: echegoyen@utep.edu; b Departament de Química Física i Inorgànica , Universitat Rovira i Virgili , C/Marcel⋅lí Domingo 1 , 43007 Tarragona , Spain; c Laboratory of Advanced Optoelectronic Materials , College of Chemistry , Chemical Engineering and Materials Science , Soochow University , Suzhou , Jiangsu 215123 , P. R. China

## Abstract

Charge transfer is a general phenomenon observed for all endohedral mono-metallofullerenes. Since the detection of the first endohedral metallofullerene (EMF), La@C_82_, in 1991, it has always been observed that the oxidation state of a given encapsulated metal is always the same, regardless of the cage size. No crystallographic data exist for any early actinide endohedrals and little is known about the oxidation states for the few compounds that have been reported. Here we report the X-ray structures of three uranium metallofullerenes, U@*D*_3h_-C_74_, U@*C*_2_(5)-C_82_ and U@*C*_2v_(9)-C_82_, and provide theoretical evidence for cage isomer dependent charge transfer states for U. Results from DFT calculations show that U@*D*_3h_-C_74_ and U@*C*_2_(5)-C_82_ have tetravalent electronic configurations corresponding to U^4+^@*D*_3h_-C_74_^4–^ and U^4+^@*C*_2_(5)-C_82_^4–^. Surprisingly, the isomeric U@*C*_2v_(9)-C_82_ has a trivalent electronic configuration corresponding to U^3+^@*C*_2v_(9)-C_82_^3–^. These are the first X-ray crystallographic structures of uranium EMFs and this is first observation of metal oxidation state dependence on carbon cage isomerism for mono-EMFs.

## Abstract 2

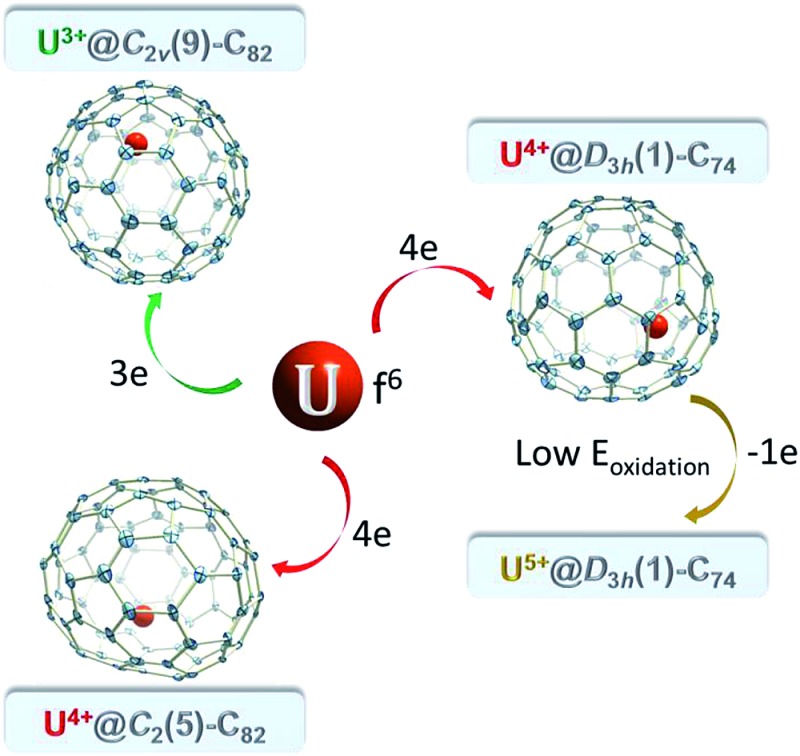
First X-ray structures and metal oxidation state dependence on cage isomerism for U-EMFs.

## Introduction

Endohedral metallofullerenes (EMFs) have attracted considerable attention due to their unique electronic properties, which arise mainly as a consequence of the intramolecular metal-to-cage charge transfer.^1^–^4^ Of these systems, mono-EMFs are particularly interesting because they represent the simplest model to probe the specific interactions between the inner metal and the outer cage. To date, many rare-earth metals and neighboring elements (groups II, III, IV) have been reported to form air-stable mono-EMFs that exhibit +2 or +3 oxidation states inside the fullerene cages.^5^–^10^ Notably, the charge of the metal ion determines the isomeric forms of the host mono-EMF cages. For instance, for C_82_-EMFs, isomers *C*_2_(5)-C_82_, *C*_s_(6)-C_82_, *C*_3v_(7)-C_82_ and *C*_2v_(9)-C_82_ containing divalent rare-earth metals have been isolated and structurally characterized,^11^–^14^ while *C*_2v_(9)-C_82_ and *C*_s_(6)-C_82_ are known to contain encapsulated trivalent lanthanides.^15^–^17^ The number of electrons that are transferred from the metal to the cage has a significant effect on the properties of mono-EMFs. For example, the divalent metallofullerenes M@C_74_ (M = Ba,^18^ Ca,^19^ Sr,^20^ Sm,^21^ Eu^22^ and Yb^23^,^24^) exhibit improved solubility relative to La@C_74_, which is almost insoluble in most common organic solvents.

Actinide EMFs represent an exciting and underexplored area of EMF chemistry. In comparison to the rare-earth elements, the early actinides (Th–Pu) are highly redox active metals possessing several accessible oxidation states. For instance, uranium oxidation states typically range from U(iii) to U(vi) while U(ii) has been recently reported.^25^–^27^ Thus, in principle, uranium offers five accessible oxidation states, in contrast with the rather limited Ln(ii)/Ln(iii) redox states of the rare-earth series. Additionally, the 5f-orbitals of the actinides are chemically accessible,^25^,^28^ contrasting the core-like 4f-orbitals of the lanthanides, suggesting potentially new EMF electronic structures and cage isomer preferences for the actinides.

Understanding the structures and electronic properties of U-EMFs is interesting and potentially significant. Perhaps due to the radioactivity of the actinide elements, the investigation of U-EMFs has generally been confined to theoretical model calculations and a few spectroscopic investigations. In 1992, Smalley and co-workers^29^ observed the formation of U@*T*_d_-C_28_. X-ray photoelectron spectroscopy (XPS) and calculations suggested a four electron uranium-to-cage charge transfer, U^4+^@C_28_^4–^. In a separate study, the electronic structure of U@C_82_ was assigned as U^3+^@C_82_^3–^ according to similarities in the UV-Vis-NIR absorptions between U@C_82_ and other M@C_82_ containing trivalent lanthanide ions.^30^ Furthermore, the X-ray absorption near edge structure (XANES) spectrum of U@C_82_ shows some similarity to that for UCl_3_, supporting a U(iii) charge assignment.^31^,^32^ The corresponding theoretical results for the molecular structure and associated electronic properties of U@C_82_ indicated that the most thermodynamically stable isomer, U@*C*_2v_(9)-C_82_, is a trivalent EMF with an electronic configuration of U^3+^@C_82_^3–^, while *C*_2_(5)-C_82_ and *C*_3v_(8)-C_82_ would favor the encapsulation of U^+^ and U^4+^, respectively.^33^

Herein we report the isolation and first single crystal X-ray crystallographic characterization of three mono-EMFs containing a single encapsulated uranium atom, namely, U@*D*_3h_-C_74_, U@*C*_2_(5)-C_82_ and U@*C*_2v_(9)-C_82_. Surprisingly, we find that the oxidation state of uranium in U@C_74_ and the two U@C_82_ isomers depends on the cage that encapsulates the U atom: U^4+^@*D*_3h_-C_74_^4–^, U^4+^@*C*_2_(5)-C_82_^4–^ and U^3+^@*C*_2v_(9)-C_82_^3–^.

## Experimental

### General instruments

HPLC separations were accomplished using a Varian Prostar instrument. Laser desorption/ionization time-of-flight (LDI-TOF) mass spectrometry was conducted on a Bruker Microflex LRF mass spectrometer. Vis-NIR spectra were obtained with a Cary 5000 UV-Vis-NIR spectrophotometer in toluene. Cyclic voltammograms (CV) and square wave voltammograms (SWV) were measured in *o*-dichlorobenzene with 0.05 M *n*-Bu_4_NPF_6_ as the supporting electrolyte using a CH Instrument Potentiostat. A 1 mm diameter glassy carbon disk was used as the working electrode, with a platinum wire and silver wire as the counter reference electrodes, respectively. All potentials were reported relative to the Fc/Fc^+^ couple.

### Synthesis and isolation of U@C_74_ and U@C_82_ (I, II)

Soot containing uranium metallofullerenes was synthesized using a direct-current arc discharge method. The raw soot was refluxed in CS_2_ for 12 h. After removal of CS_2_, the residue was dissolved in toluene and the solution was subjected to a multi-stage HPLC separation process. Further details are described in the ESI.†

### Single-crystal X-ray diffraction

Crystalline blocks of U@*C*_2*n*_ (2*n* = 74, 82) were obtained by layering a benzene solution of Ni^II^(OEP) over a nearly saturated solution of the endohedral in CS_2_ in a glass tube. Over a 20 day period, the two solutions diffused into each other and black crystals formed. XRD measurements were performed at 150 K on a Bruker P4 machine equipped with a graphite monochromator. The multi-scan method was used for absorption corrections. The structures were solved by a direct method and were refined with SHELXL-2013.^34^

Crystal data for U@*D*_3h_-C_74_·Ni^II^(OEP)·2(toluene): C_122_H_56_N_4_NiU, *M*_w_ = 1874.28, monoclinic, space group *C*2/*c*, *a* = 25.102(3) Å, *b* = 14.9208(19) Å, *c* = 38.636(5) Å, *β* = 94.044(2)°, *V* = 14 435(3) Å^3^, *Z* = 8, *T* = 150 K, *ρ*_calcd_ = 1.725 Mg m^–3^, *μ*(MoKα) = 2.569 mm^–1^, 72 450 reflections measured, 18 022 unique (*R*_int_ = 0.0981) used in all calculations. The final w*R*_2_ was 0.3355 (all data) and *R*_1_ (11 937 with *I* > 2\*s*(*I*)) = 0.1247. The relatively high *R*_1_ and w*R*_2_ values are due to the severe disorder in the cage, the metal atom and the intercalated solvent molecules. CCDC ; 1508508 contains the crystallographic data.†

Crystal data for U@*C*_2_(5)-C_82_·Ni^II^(OEP)·2(toluene): C_130_H_56_N_4_NiU, *M*_w_ = 1970.37, monoclinic, space group *C*2/*m*, *a* = 25.0308(10) Å, *b* = 15.3288(6) Å, *c* = 19.9410(8) Å, *V* = 7632.7(5) Å^3^, *Z* = 4, *T* = 150 K, *ρ*_calcd_ = 1.715 Mg m^–3^, *μ*(MoKα) = 2.434 mm^–1^, 42 398 reflections measured, 9067 unique (*R*_int_ = 0.0328) used in all calculations. The final w*R*_2_ was 0.2290 (all data) and *R*_1_ (7016 with *I* > 2\*s*(*I*)) = 0.0779. CCDC ; 1508509 contains the crystallographic data.†

Crystal data for U@*C*_2v_(9)-C_82_·Ni^II^(OEP)·1.5(toluene)·CS_2_: C_128_H_53_N_4_NiS_2_U, *M*_w_ = 2007.56, monoclinic, space group *P*2_1_/*c*, *a* = 17.7846(5) Å, *b* = 17.2807(5) Å, *c* = 26.6533(7) Å, *V* = 7817.6(4) Å^3^, *Z* = 4, *T* = 150 K, *ρ*_calcd_ = 1.706 Mg m^–3^, *μ*(MoKα) = 2.431 mm^–1^, 96 891 reflections measured, 14 336 unique (*R*_int_ = 0.0840) used in all calculations. The final w*R*_2_ was 0.2849 (all data) and *R*_1_ (9344 with *I* > 2\*s*(*I*)) = 0.0987. CCDC ; 1522558 contains the crystallographic data.†

### Computational details

All calculations were carried out using density functional theory (DFT) with the ADF 2013 package.^35^ The exchange-correlation functionals of Becke and Perdew (BP86) were used.^36^,^37^ Slater triple-zeta polarization (TZP) basis sets were used to describe the valence electrons of Ba, La, Hf, U and C. Frozen cores were described by means of single Slater functions, consisting of the 1s shell for C, the 1s to 5d shells for U, 1s to 4d shells for La and Hf, and 1s to 4p shells for Ba. Scalar and spin–orbit relativistic corrections were included by means of the ZORA formalism. Open-shell calculations were performed at an unrestricted level. For the spin–orbit calculations, we carried out single-point energy calculations at the geometry optimized at BP86/TZP + scalar relativistic level, with a basis set of TZ2P quality with no core for U. Electrochemistry calculations were performed at the same level of theory BP86/TZP, with dichlorobenzene as solvent. A data set collection of computational results is available in the ioChem-BD repository and can be accessed *via*; http://doi.org/10.19061/iochem-bd-2-14.^38^

## Results and discussion

### Characterization of U@*C*_2*n*_ (2*n* = 74, 82)

**Fig. 1:**
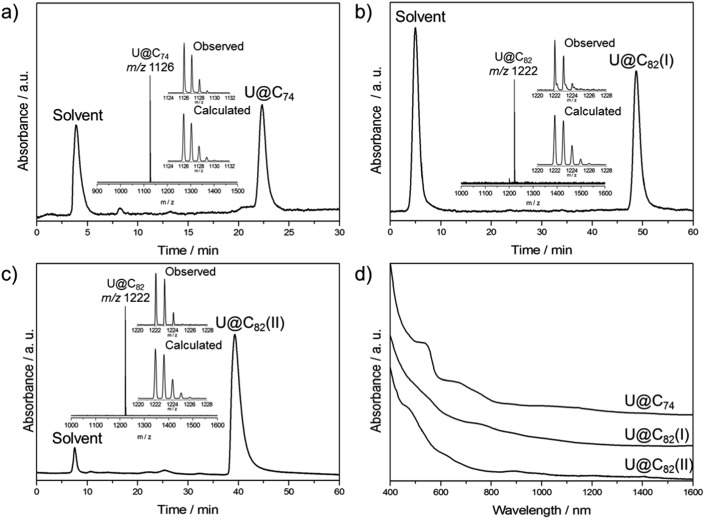
HPLC chromatograms of purified (a) U@C_74_, (b) U@C_82_ (I) and (c) U@C_82_ (II) on a Buckyprep column with toluene as the eluent (HPLC conditions: flow rate, 1.0 mL min^–1^; detection wavelength, 330 nm. Insets show the LDI-TOF mass spectra and expansions of the observed isotopic distributions of U@C_74_ and U@C_82_ in comparison with the calculated ones, respectively). (d) Vis-NIR absorption spectra of purified U@C_74_ and U@C_82_ (I, II) in toluene.

Soot containing uranium endohedral fullerenes was obtained from graphite rods filled with uranium oxide and graphite powder (weight ratio of U/C = 1 : 4) in a conventional Krätschmer-Huffman arc-discharge reactor under a 200 Torr helium atmosphere.^39^ The as-produced fullerene soot was Soxhlet-extracted with carbon disulfide (CS_2_) for 24 h. Multi-stage HPLC separations gave pure isomers of U@C_74_ and U@C_82_ (I, II) (see Fig. S1–S4†). Fig. 1 shows the chromatograms and mass spectra of the purified samples. The Vis-NIR spectra of the three compounds are shown in Fig. 1d. Their spectral onsets are located at around 1500 nm and 1300 nm, respectively, thus reflecting rather small HOMO–LUMO gaps. U@C_74_ displays absorption bands at 1399, 1018, 878, 743, 672 and 538 nm, U@C_82_ (I) exhibits distinct absorptions at 885, 760 and 535 nm, and U@C_82_ (II) exhibits distinct absorptions at 1405, 1204, 1003, 893, 620 and 469 nm.

### Single-crystal X-ray structure of U@*D*_3h_-C_74_, U@*C*_2_(5)-C_82_ and U@*C*_2v_(9)-C_82_

Single crystals of U@C_74_ and U@*C*_82_ (I, II) were obtained by layering a benzene solution of Ni^II^(OEP) (OEP = 2,3,7,8,12,13,17,18-octaethylporphyrin dianion) over a nearly saturated solution of the EMFs in CS_2_ in a glass tube. The molecular structures of U@C_74_ and U@C_82_ (I, II) were unambiguously determined using single crystal X-ray diffraction. Analysis of cage connectivity reveals the cage isomers *D*_3h_-C_74_, *C*_2_(5)-C_82_ and *C*_2v_(9)-C_82_ (nomenclature in accordance with the spiro algorithm).^40^ Notably, the above-mentioned Vis-NIR spectra of the first two compounds are substantially different from those of crystallographically characterized endohedral fullerenes possessing the same cage, such as Sm@*D*_3h_-C_74_,^21^ Ba@*D*_3h_-C_74_,^18^ and Sm@*C*_2_(5)-C_82_,^11^ whereas the absorption features of U@*C*_2v_(9)-C_82_ are similar to those of La@*C*_2v_(9)-C_82_. These results suggest that U@*D*_3h_-C_74_ and U@*C*_2_(5)-C_82_ have formal charge transfers different (likely larger) from +2 while U@*C*_2v_(9)-C_82_ likely involves a +3 charge transfer, as observed for La^3+^.^16^

**Fig. 2:**
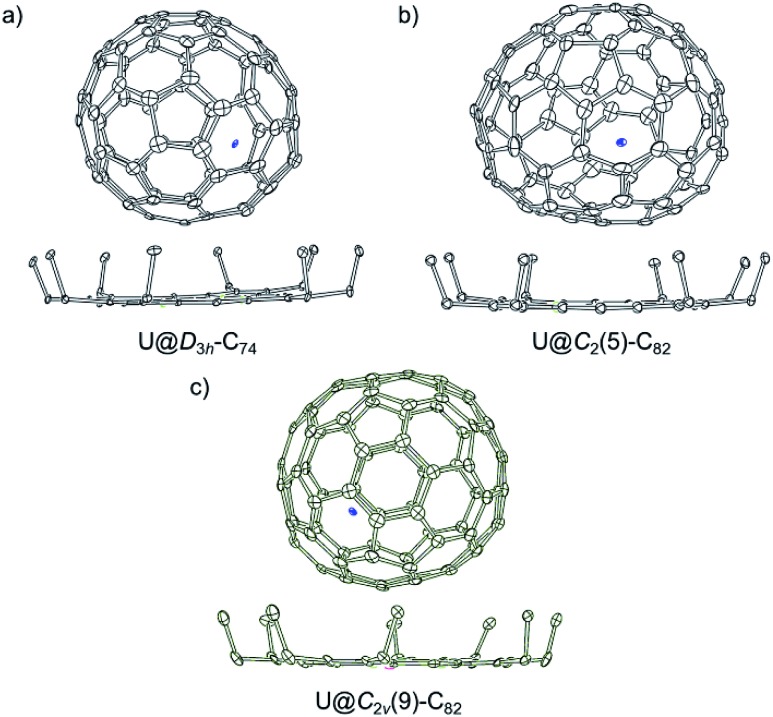
ORTEP drawings showing the relative orientations of the uranium endohedrals and porphyrin for (a) U@*D*_3h_-C_74_·Ni^II^(OEP)·2(toluene), (b) U@*C*_2_(5)-C_82_·Ni^II^(OEP)·2(toluene) and (c) U@*C*_2v_(9)-C_82_·Ni^II^(OEP)·1.5(toluene)·CS_2_. Thermal ellipsoids are shown at the 10% probability level. Only the major fullerene cage and the predominant uranium orientation are shown, and minor sites and solvent molecules are omitted for clarity.

**Fig. 3:**
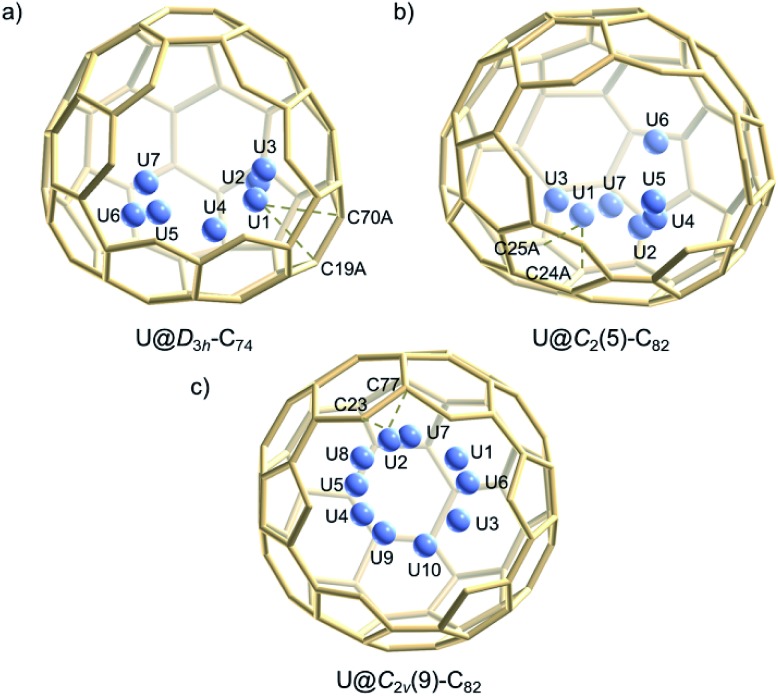
Perspective drawings show seven positions of the disordered uranium sites in (a) U@*D*_3h_-C_74_, (b) U@*C*_2_(5)-C_82_ and (c) ten positions in U@*C*_2v_(9)-C_82_. The 3-fold axis of *D*_3h_-C_74_ is perpendicular to the plane, while the 2-fold axes of *C*_2_(5)-C_82_ and *C*_2v_(9)-C_82_ are aligned vertically.

Fig. 2 shows the single crystal X-ray structure of U@*D*_3h_-C_74_, U@*C*_2_(5)-C_82_ and U@*C*_2v_(9)-C_82_ relative to Ni^II^(OEP). The porphyrin moiety faces a flat region for the three systems *D*_3h_-C_74_, *C*_2_(5)-C_82_ and *C*_2v_(9)-C_82_, with the shortest nickel-to-cage carbon distance ranging from 2.787 to 2.929 Å, indicative of strong π–π stacking interactions. The U ions in all three compounds show some degree of disorder, reflecting restricted motion of the U atom within the cage. Fig. 3 shows the positions of the disordered U atoms. For all three compounds, the U ions are highly localized near one part of the molecule. For U@*D*_3h_-C_74_, seven U ion positions have been assigned, with fractional occupancies ranging from 0.01 to 0.46. Similarly, there are also seven U ion positions with fractional occupancies that range from 0.02 to 0.26 for U@*C*_2_(5)-C_82_. Ten different positions, which are arranged in a circle, are found for the U atom in U@*C*_2v_(9)-C_82_. These disorder features are quite similar to those observed for mono-metallofullerenes encapsulating Group II, Group III and lanthanide metals. The predominant U position with fractional occupancies of 0.46 in U@*D*_3h_-C_74_ is situated over the central [6,6]-bond of one of the three symmetry-equivalent pyracylene units (Fig. S5a†). Somewhat surprisingly, similar arrangements have been reported previously for two other divalent mono metallofullerenes: Sm^2+^@*D*_3h_-C_74_^2–^ and Ba^2+^@*D*_3h_-C_74_^2–^.^18^,^21^ The major U position for U@*C*_2_(5)-C_82_ (with 0.26 fractional occupancy) is situated over a [5,6]-bond and is identical to the mirror-related major Sm site for Sm^2+^@*C*_2_(5)-C_82_^2–^ (Fig. S5b†).^11^ The U ion in U@*C*_2v_(9)-C_82_ (with 0.22 fractional occupancy) is situated over the center of a hexagon that radiates from the *C*_2_ axis, which differs from that of the Sm^2+^ atom in Sm@*C*_2v_(9)-C_82_ but is very close to the La^3+^ site in La^3+^@*C*_2v_(9)-C_82_^2–^ (Fig. S5c†). Considering the spectroscopic similarities between U@*C*_2v_(9)-C_82_ and La@*C*_2v_(9)-C_82_,^16^ it is reasonable to speculate that the oxidation state of the U atom in *C*_2v_(9)-C_82_ is +3. However, the most populated sites of the U ion in *D*_3h_-C_74_ and *C*_2_(5)-C_82_ are similar to those observed for divalent metallofullerenes to some extent, but their corresponding Vis-NIR spectra are totally different. Such inconsistencies require reconsideration of the electronic structures of U@*D*_3h_-C_74_ and U@*C*_2_(5)-C_82_, which seem to be somewhat unique.

### In-depth analysis of the electronic structures of U-EMFs

Without exception, the oxidation state of any given encapsulated metal atom (Group-2, Group-3 or lanthanides) in mono-EMFs is always the same regardless of the size or geometry of the corresponding fullerene cages. Based on the electronic absorption spectra and solid-state structures of the three uranium mono-EMFs, it seems that the oxidation state of uranium can depend on the cage isomer. In order to gain insight about the uranium charge oxidation states for the three uranium mono-EMFs we performed DFT calculations at the BP86/TZP level, taking into account relativistic effects (scalar relativistic as well as spin–orbit coupling, see ESI for more details†), which are of major importance for actinides. Below, we show for the first time that the cage isomer plays a critical role in determining the corresponding ground-state electronic configuration of U-EMFs.

**Fig. 4:**
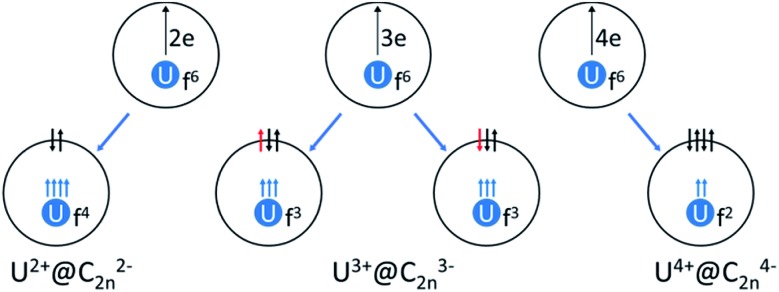
Schematic representation of the electronic structure of U^2+^@*C*_2*n*_^2–^ (quintet state), U^3+^@*C*_2*n*_^3–^ (two different spin multiplicities: quintet or triplet) and U^4+^@*C*_2*n*_^4–^ (triplet state).

**Fig. 5:**
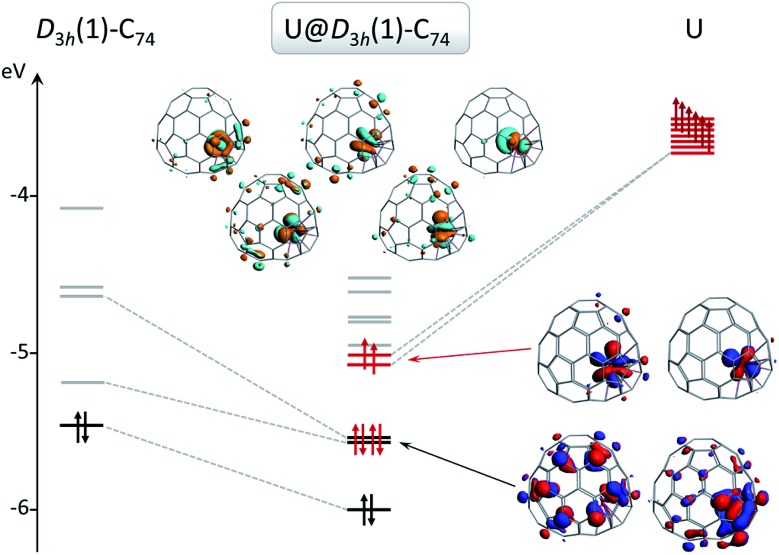
Representation of the orbital interaction diagram between uranium and the C_74_ cage for U@*D*_3h_-C_74_ in the triplet state. Electrons from the cage and uranium are represented as black and red arrows, respectively. The same colors are used for the occupied orbitals (black for the cage and red for U) while empty orbitals are represented in grey.

On the basis of the ionic model,^41^–^43^ different possible situations could exist for the mono-EMFs, the most likely ones being: U^2+^@*C*_2*n*_^2–^, U^3+^@*C*_2*n*_^3–^ or U^4+^@*C*_2*n*_^4–^ (Fig. 4). We have not considered explicitly oxidation states for U larger than four because the amount of charge transferred in medium-size mono-EMF has never been observed to exceed three. We have computed U@*D*_3h_-C_74_ in triplet and quintet states, and the triplet is 8.3 kcal mol^–1^ lower in energy than the quintet. The optimized structures for both multiplicities are essentially the same, with the uranium located in one of the crystallographically determined sites exhibiting a high occupancy value. To probe the electron transfer properties we analyzed the molecular orbital diagram of U@*D*_3h_-C_74_ in the triplet state (Fig. 5). Although calculations were carried out at an unrestricted level in Fig. 5, we preferred not to differentiate between alpha and beta orbitals, and a schematic representation is presented for simplicity. The four highest-energy electrons of the uranium are transferred to those unoccupied orbitals of the fullerene cage with lowest energies. Consequently, only two electrons remain in the uranium f-orbitals, a fact that is confirmed by the Mulliken spin density of the uranium atom, which is close to 2. Therefore, the electronic structure can be represented within the ionic model as U^4+^@*D*_3h_-C_74_^4–^. For the quintet state, however, we found a three-electron transfer with one unpaired electron on the cage ferromagnetically coupled with the three 5f uranium electrons.

For the C_82_ cage, out of 39 718 isomers, there are only nine that satisfy the isolated pentagon rule (IPR). In general, it is rather useful to analyze the neutral and anionic species to estimate the ability of the cage to encapsulate metals in different oxidation states. Indeed, Liu *et al.*^33^ performed an exhaustive study of the ionic forms of C_82_ cages concluding that (1) IPR isomers are, in general, much more stable than the non-IPR forms with a pentalene (*i.e.* one adjacent pentagon pair, APP1) and (2) *C*_2_(5)-C_82_ and *C*_3v_(8)-C_82_ are the best cages for the encapsulation of monovalent and tetravalent cations, respectively, whereas *C*_2v_(9)-C_82_ is the optimal cage to encapsulate M^2+^, M^3+^, M^5+^and M^6+^ species. It is worth mentioning that *C*_2_(5)-C_82_ was not found as an optimal cage to encapsulate a tetravalent ion but rather a monovalent species.

**Fig. 6:**
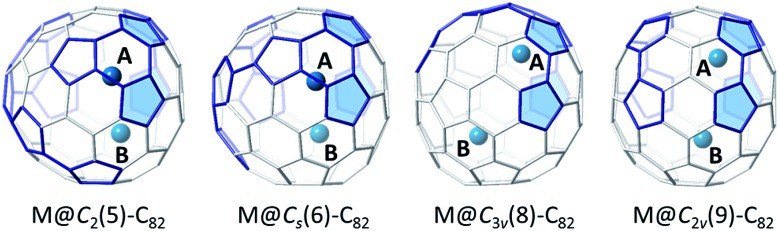
Structures for the EMFs M@C_82_ (M = Ba, La, Hf, U) showing positions A and B of the metal atom for M@*C*_2_(5)-C_82_, M@C_s_(6)-C_82_, M@*C*_3v_(8)-C_82_ and M@*C*_2v_(9)-C_82_. Bonds forming pyracylene units are highlighted in blue. Pentagons of pyracylene units fixed at the same position are filled in blue.

Based on the known oxidation states of uranium, U^+^ encapsulated inside a fullerene cage is highly unlikely. To determine the likely oxidation states for the U@C_82_ endohedrals we performed a series of calculations for cages #5, #6, #8 and #9 using U as the encapsulated atom, and also with Ba, La and Hf, which are useful models for two, three and four electron transfers between the captured atom and the carbon cage. These four C_82_ isomers are related by Stone–Wales (SW) transformations (see Fig. S6†). One structural pattern that is conserved in these four isomers is the chain of three pyracylene units (four pentagons) depicted at the right of cage #9 in Fig. 6. Taking this pattern as the reference, we oriented the cages so that the pyracylene units are fixed in the same position. We have then considered metal ions encapsulated in these four cages at two different positions A (at the top) and B (at the bottom), following the work of Liu *et al.*^33^ We have verified that other starting positions collapse to one of these two sites A or B.

As shown in Table 1 the relative stabilities of dianionic, trianionic, and tetraanionic cages match rather well with those of endohedral models containing Ba, La and Hf when the cation is located in site B. Remarkably, cage #5 seems to have the dual ability of stabilizing divalent cations in the bottom hemisphere (site B), or tetravalent cations in the top hemisphere (site A). For La^3+^, there is no preferential region inside this cage (see Table 1).

**Table 1:** Relative energies for several M@C_82_ endohedrals with different amount of charge transferred^*a*^

Isomer	C_82_^2–^	C_82_^3–^	C_82_^4–^	Site^*b*^	Ba@C_82_ (singlet)	La@C_82_ (singlet)	Hf@C_82_ (singlet)	U@C_82_ (triplet)	U@C_82_ (quintet)
*C*_2_(5)-C_82_	3.9	11.1	22.3	B	5.0	13.1	17.6	15.3	14.7
				A	—	14.7	5.0	1.9^*c*^	11.0
*C*_s_(6)-C_82_	2.7	3.5	8.4	B	2.4	4.9	6.0	4.7	4.8
				A	—	17.9	12.8	8.0	12.5
*C*_3v_(8)-C_82_	8.8	2.9	0.0	B	10.8	4.8	0.0	2.2^*c*^	5.0
				A	31.8	31.5	30.2	25.3	20.9
*C*_2v_(9)-C_82_	0.0	0.0	3.0	B	0.0	0.0	0.0	0.0	0.6
				A	21.4	29.9	—	28.3	25.1

^*a*^Energies are in kcal mol^–1^.

^*b*^Computed positions for the metal ion (see Fig. 7).

^*c*^Charge transfer for U@C_82_ endohedral fullerenes is 3+ except for U@*C*_2_(5)-C_82_ site A and for U@*C*_3v_(8)-C_82_ site B (triplet) for which it is 4+ (see text for more details).

We proceeded to study in detail the electronic structure of U@*C*_2_(5)-C_82_, U@*C*_s_(6)-C_82_, U@*C*_3v_(8)-C_82_ and U@*C*_2v_(9)-C_82_ for triplet and quintet states (Fig. 4). The energy difference between these two states is in general small, with the triplet state favored over the quintet (see Table 1). U@*C*_2v_(9)-C_82_ B and U@*C*_2_(5)-C_82_ A are the lowest-energy isomers in good agreement with experiments, and the uranium sites predicted by the calculations correspond to the positions with major occupancy factors derived from the crystallographic structures (see Fig. S7†). We have checked that the relative energies computed so far are essentially kept (within 1.5 kcal mol^–1^) once the spin–orbit corrections are introduced in the calculations (see Table S1†). Relative energies for U@C_82_ in the triplet state with the guest ion located in site B correlate rather well with energies computed for the trianionic cages. For U@*C*_2_(5)-C_82_ we can observe a similar behavior to that found for Hf@*C*_2_(5)-C_82_, resulting in position A being more favored than B. These results clearly suggest that the oxidation state of uranium is U^4+^ for U@*C*_2_(5)-C_82_ and likely U^3+^ or U^4+^ for the other three cages.

**Fig. 7:**
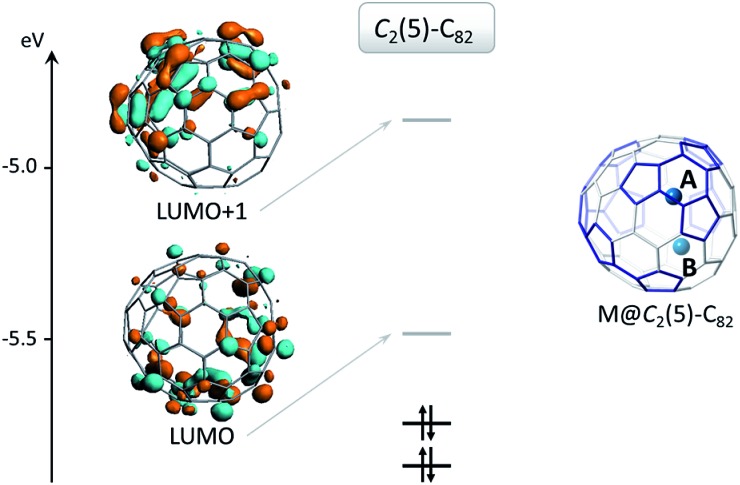
Molecular orbital diagram for isolated cage *C*_2_(5)-C_82_. The LUMO is occupied when the cage captures a divalent cation and the LUMO and LUMO+1 are occupied when the cation is tetravalent. The figure also shows preferential locations for divalent (site B) and tetravalent (site A) cations.

To understand the electronic structure of U@*C*_2_(5)-C_82_ and U@*C*_2v_(9)-C_82_, we analyzed the molecular orbital diagram in the same way as for U@*D*_3h_-C_74_. For U@*C*_2_(5)-C_82_ in the triplet state (see Fig. S8†), there is a formal transfer of four electrons from uranium to the fullerene cage with a spin density close to 2 for the uranium atom as for U@*D*_3h_-C_74_. For the quintet state we also found a four-electron transfer. For U@*C*_2v_(9)-C_82_, we have seen that triplet and quintet states are almost degenerate. In Fig. S8† we present the molecular orbital diagram of the quintet state, since it is easier to interpret than that for the triplet. Interestingly, only three electrons are transferred to the cage, instead of four as in U@*D*_3h_-C_74_ and U@*C*_2_(5)-C_82_. The spin density of uranium is close to 3 in this case. For the triplet state, an intermediate situation between a three and a four-electron transfer is observed. Therefore, for U@*C*_2v_(9)-C_82_, the oxidation state of uranium is closer to three than four. To conclude, the degree of charge transfer for U@C_82_ isomers depends on the specific cage that encapsulates the U atom: U^4+^@*C*_2_(5)-C_82_^4–^ and U^3+^@*C*_2v_(9)-C_82_^3–^. For the other two computed isomers, not yet experimentally obtained, we have U^3+^@*C*_s_(6)-C_82_^3–^ and U^4+^@*C*_3v_(8)-C_82_^4–^ for the triplet state, which changes to U^3+^@*C*_3v_(8)-C_82_^3–^ for the energetically accessible quintet state (only 2.5 kcal mol^–1^ higher in energy, see Table 1). These results are consistent with previous experimental observations where cage *C*_2v_(9)-C_82_ was found to encapsulate M^3+^ ions whereas cages *C*_s_(6)-C_82_ and *C*_3v_(8)-C_82_ preferentially encapsulate tetravalent clusters (M_2_O, M_2_S and M_2_C_2_).^44^–^48^ It is interesting to point out that even though the C_74_ and C_82_ cages that encapsulate Sm and Yb are the same as the two found for U, M^2+^@*D*_3h_(1)-C_74_^2–^ and M^2+^@*C*_2_(5)-C_82_^2–^, we have not observed a transfer of two electrons for any of the U-EMFs analyzed here. The reason for this apparent molecular electronic promiscuity is that *D*_3h_(1)-C_74_ and *C*_2_(5)-C_82_ cages are able to stabilize both 4 electron (U) as well 2 electron transfers (Sm and Yb). For example, for cage *C*_2_(5)-C_82_, the bottom hemisphere location is optimal for hosting M^2+^ (site B) whereas M^4+^ preferentially locate at site A. When a divalent metal ion is encapsulated, two electrons are transferred from the metal to the LUMO of the fullerene cage, which is mainly located at the bottom, as shown in Fig. 7. Consequently, the metal-cage interaction is optimal when the metal atom is located in this hemisphere. Alternatively, when the *C*_2_(5)-C_82_ cage encapsulates a tetravalent cation the LUMO and LUMO+1 are occupied and in this case the optimal metal-carbon cage interactions occur when the metal ion is near site A at the top hemisphere region where the LUMO+1 is mainly localized.

**Fig. 8:**
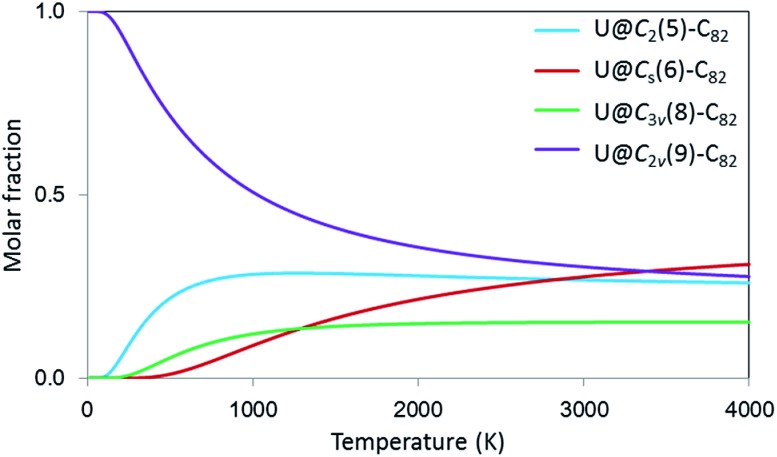
Computed molar fraction as a function of the temperature (K) using the free-encapsulating model (FEM) for U@*C*_2_(5)-C_82_, U@*C*_s_(6)-C_82_, U@*C*_3v_(8)-C_82_ and U@*C*_2v_(9)-C_82_.

Finally, to incorporate the effect of the high temperatures at which the fullerenes are formed, which can be critical to determine their relative stabilities and abundances,^48^ we have also computed the molar fractions of these four isomers as a function of temperature (0–4000 K) using the rigid rotor and harmonic approximation (RRHO) and the related free-encapsulating model (FEM) as proposed by Slanina.^49^,^50^ Fig. 8 shows molar fractions using the FEM approximation, where U@*C*_2v_(9)-C_82_ is the most abundant up to 3000 K followed by U@*C*_2_(5)-C_82_ in good agreement with experimental results. Isomers U@*C*_s_(6)-C_82_ and U@*C*_3v_(8)-C_82_, which also show appreciable molar fractions at high temperatures, might also be detected in future experiments.

### Electrochemical properties of U^4+^@*D*_3h_-C_74_^4–^, U^4+^@*C*_2_(5)-C_82_^4–^ and U^3+^@*C*_2v_(9)-C_82_^3–^

**Fig. 9:**
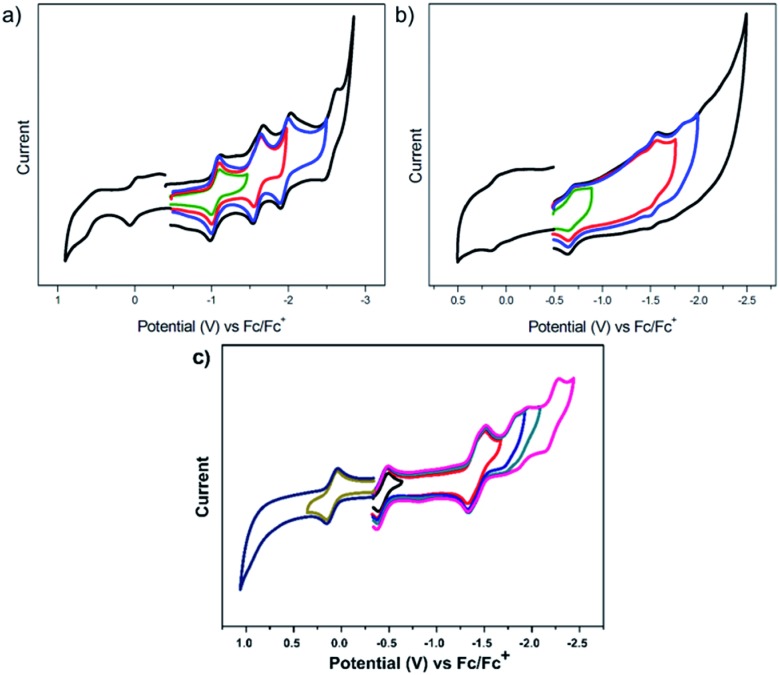
Cyclic voltammograms of U@*D*_3h_-C_74_ (a), U@*C*_2_(5)-C_82_ (b) and U@*C*_2v_(9)-C_82_ (c) in 0.05 M *n*-Bu_4_NPF_6_/*o*-DCB solution, (scan rate: 20 mV s^–1^).

The redox properties of U^4+^@*D*_3h_-C_74_^4–^, U^4+^@*C*_2_(5)-C_82_^4–^ and U^3+^@*C*_2v_(9)-C_82_^3–^ were measured by cyclic voltammetry (CV) (Fig. 9) and square wave voltammetry (SWV) (see Fig. S9†) in *o*-dichlorobenzene (*o*-DCB) solution containing 0.05 M *n*-Bu_4_NPF_6_ as the supporting electrolyte. Four reversible one-electron reductive steps with nearly equal distance and two reversible one-electron oxidative steps were observed for U^4+^@*D*_3h_-C_74_^4–^. Interestingly, despite the different metal-to-cage charge transfer, the redox behavior of U^4+^@*D*_3h_-C_74_^4–^shows remarkable resemblance to those reported for Sm^2+^@*D*_3h_-C_74_^2–^ and Eu^2+^@*D*_3h_-C_74_^2–^, which also exhibit four reversible reductive steps and two reversible oxidative steps.^21^,^22^ In addition, the electrochemical band gap of U^4+^@*D*_3h_-C_74_^4–^ (1.06 eV) is also very close to those of the Sm^2+^@*D*_3h_-C_74_^2–^ (0.97 eV) and Eu^2+^@*D*_3h_-C_74_^2–^ (1.00 eV), a clear indication of their common closed-shell electronic structures. However, despite the similarity in the number and regularity of the redox steps, it is worth mentioning that all potential values for U^4+^@*D*_3h_-C_74_^4–^ are markedly shifted cathodically (by 200 to 400 mV), indicating that it is much easier to oxidize and more difficult to reduce than the corresponding Sm^2+^@*D*_3h_-C_74_^2–^. This likely due to the much higher negative charge localized on the cage for U^4+^@*D*_3h_-C_74_^4–^.

For the larger C_82_ cages, specifically for U^4+^@*C*_2_(5)-C_82_^4–^, the electrochemical behavior is very different when compared with those of other mono-metallofullerenes with the same *C*_2_(5)-C_82_ cage. U^4+^@*C*_2_(5)-C_82_^4–^ exhibits two reversible and two irreversible one-electron reductive processes, whereas all of the one-electron reductive processes of Yb^2+^@*C*_2_(5)-C_82_^2–^ and Sm^2+^@*C*_2_(5)-C_82_^2–^ are perfectly reversible.^12^,^51^ As expected, the overall redox behavior of U^3+^@*C*_2v_(9)-C_82_^3–^ shows remarkable resemblance to those of other M^3+^@*C*_2v_(9)-C_82_^3–^ (M = La, Y, Ce),^52^,^53^ while it is very different compared with those of divalent mono-EMFs (*e.g.* Yb^2+^@*C*_2v_(9)-C_82_^2–^, Sm^2+^@*C*_2v_(9)-C_82_^2–^).^12^,^14^ Not surprisingly, the degree of metal-to-cage charge transfer strongly influences the electrochemical properties of mono-EMFs as well as the isomeric structure. Remarkably, despite the size and structural diversity of the cages, all U-EMFs share one common electrochemical feature: they are all reasonably easy to oxidize 0.01 V (U@*D*_3h_-C_74_), 0.11 V (U@*C*_2_(5)-C_82_) and 0.10 V (U^3+^@*C*_2v_(9)-C_82_^3–^), making them reasonably good electron-donors (Table 2).

We also computed the first reduction and oxidation potentials for U^4+^@*D*_3h_-C_74_^4–^, U^4+^@*C*_2_(5)-C_82_^4–^ and U^3+^@*C*_2v_(9)-C_82_^3–^, in order to compare with experimental results (see Table S2†). The computed potentials are comparable to the experimental values within the error of the methodology,^54^ although the deviation of the electrochemical gap for U^4+^@*D*_3h_-C_74_^4–^ was larger than for the other uranium fullerenes. After a detailed analysis of the molecular orbitals involved in the redox processes we concluded that for U^4+^@*D*_3h_-C_74_^4–^ and U^4+^@*C*_2_(5)-C_82_^4–^ reduction takes place on uranium, whereas for U^3+^@*C*_2v_(9)-C_82_^3–^ reduction occurs on the carbon cage. Oxidation processes are rather specific for each case. Hence, we have found that U(iii) is oxidized to U(iv) when it is encapsulated by *C*_2v_(9)-C_82_, and U(iv) oxidizes up to U(v) in the smaller cage C_74_. However, the first oxidation of U^4+^@*C*_2_(5)-C_82_^4–^ occurs at the carbon cage. It is essential to remark that in some cases after oxidation or reduction several states remain close in energy. For example, the first reduction of U@*C*_2v_(9)-C_82_ at the uranium ion or on the carbon cage have similar reduction potentials. This may explain why the experimental first reduction potentials for U@*C*_2v_(9)-C_82_ and La@*C*_2v_(9)-C_82_ are similar. Estimated oxidation states for neutral and ionic species are compiled in Table S3.† Finally, we would like to point out that the monoconfigurational DFT description of these quasidegenerated states is an approximation to the real multiconfigurational wavefunction and this might be the origin of the larger discrepancies found in the estimation of the potentials and electrochemical gap for U@*D*_3h_-C_74_ compared to other urano- or clusterfullerenes.

**Table 2:** Redox potentials (V *vs.* Fc/Fc^+^)^*a*^ and electrochemical bandgaps of U@*D*_3h_-C_74_, U@*C*_2_(5)-C_82_, U@*C*_2v_(9)-C_82_ and reference endohedrals

Species	^ox^ *E*_2_ [V]	^ox^ *E*_1_ [V]	^red^ *E*_1_ [V]	^red^ *E*_2_ [V]	^red^ *E*_3_ [V]	^red^ *E*_4_ [V]	^red^ *E*_5_ [V]	Δ*E*_gap_ [V]
Sm@*D*_3h_-C_74_ (ref. 21)	0.76	0.20	–0.77	–1.21	–1.72	–2.14	—	0.97
U@*D*_3h_-C_74_	0.61	0.01	–1.05	–1.60	–1.96	–2.55	—	1.06
Sm@*C*_2_(5)-C_82_ (ref. 51)	—	0.42	–0.84	–1.01	–1.51	–1.90	—	1.26
Yb@*C*_2_(5)-C_82_ (ref. 12)	0.90	0.38	–0.86	–0.98	–1.50	–1.87	—	1.24
U@*C*_2_(5)-C_82_	—	0.11	–0.67	–1.54	–1.83^*b*^	–2.05^*b*^	—	0.78
Sm@*C*_2v_(9)-C_82_ (ref. 14)	—	0.52	–0.42	–0.77	–1.60	–1.94	—	0.94
Yb@*C*_2v_(9)-C_82_ (ref. 12)	—	0.61	–0.46	–0.78	–1.60	–1.90	—	1.07
La@*C*_2v_(9)-C_82_ (ref. 53)	1.07	0.07	–0.42	–1.37	–1.53	–2.26	—	0.49
U@*C*_2v_(9)-C_82_	0.92	0.10	–0.43	–1.42	–1.76^*b*^	–1.77^*b*^	–2.21^*b*^	0.53

^*a*^Half-cell potentials are given unless otherwise addressed.

^*b*^Irreversible. Square wave voltammetry peak values.

## Conclusions

In this work, single crystal X-ray structures and theoretical calculations of three U-EMFs, U@*D*_3h_-C_74_, U@*C*_2_(5)-C_82_ and U@*C*_2v_(9)-C_82_, reveal that a variable valence state of the encapsulated metal atom can result from isomeric fullerene cages. Notably, we find that the oxidation state of uranium for U@C_74_ and U@C_82_ isomers depend on the cage structures that encapsulates the U atom: U^4+^@*D*_3h_-C_74_^4–^, U^4+^@*C*_2_(5)-C_82_^4–^ and U^3+^@*C*_2v_(9)-C_82_^3–^. Formal transfers of two or six electrons, U^2+^@*C*_2*n*_^2–^ or U^6+^@*C*_2*n*_^6–^, are not observed for any of the systems analyzed here. The first two compounds are the first examples of tetravalent (M^4+^) containing mono-EMFs and the two U@C_82_ isomers represent the first pair where the oxidation state of the encapsulated ion depends on the isomeric cage structure (4+ or 3+). Detailed analyses further reveal that reduction takes place at the endohedral metal ion when the oxidation state of U is IV and on the fullerene cage when it is III. We also present the first single-crystal X-ray crystallographic structures for U endohedrals.

## Supplementary Material

Supplementary information

Crystal structure data
