# Supplementary material for: Single crystal structures and theoretical calculations of uranium endohedral metallofullerenes (U@C2n, 2n = 74, 82) show cage isomer dependent oxidation states for U
Source: Chem Sci. 2017 May 22;8(8):5282–90. doi: 10.1039/c7sc01711a (PMC5607891; doi:10.1039/c7sc01711a)
Supplement: Supplementary file 1 [file SC-008-C7SC01711A-s001.pdf]

Electronic Supplementary Material (ESI) for Chemical Science.
This journal is © The Royal Society of Chemistry 2017

Supporting Information of

Single Crystal Structures and Theoretical Calculations of Uranium Endohedral Metallofullerenes (U@C_{2n}, n=37, 41) Show Cage Isomer Dependent Oxidation States for U

Wenting Cai,[a] Roser Morales-Martínez,[b] Xingxing Zhang,[c] Daniel Najera,[a] Elkin L. Romero,[a] Alejandro Metta-Magaña,[a] Antonio Rodríguez-Fortea,[b] Skye Fortier,[‡] Ning Chen,*[c] Josep M. Poblet,*[b] and Luis Echegoyen*[a]

[a] Department of Chemistry, University of Texas at El Paso, 500 W University Avenue, El Paso, Texas 79968, United States.

[b] Departament de Química Física i Inorgànica, Universitat Rovira i Virgili, C/Marcel·lí Domingo 1, 43007 Tarragona (Spain)

[c] Laboratory of Advanced Optoelectronic Materials, College of Chemistry, Chemical Engineering and Materials Science, Soochow University, Suzhou, Jiangsu, 215123 (P. R. China)

Email: echegoyen@utep.edu; josepmaria.poblet@urv.cat; chenning@suda.edu.cn

Experimental details:

HPLC separation of U@D_{3h}-C_{74}, U@C_2(5)-C_{82} and U@C_{2v}(9)-C_{82}. The first stage was performed on a 5PYE column (10 mm × 250 mm, Cosmosil Nacalai Tesque) with toluene as mobile phase. Figure S1 shows the first stage HPLC chromatogram of extract. Three fractions, which were named as A, B and C, was collected, respectively. After that, fraction A was then injected into a BPD column (10 mm × 250 mm, Cosmosil Nacalai Tesque) for the second stage separation using toluene as the eluent. The fraction marked with A1, which contained U@C_{74} was collected (Figure S2a). The third stage separation for

A1 was carried out using a BP column (10 mm × 250 mm, Cosmosil Nacalai Tesque) with a toluene mobile phase, and pure U@C_{74} was finally obtained (Figure S2b). Meanwhile, fraction B was re-injected into a BPM column (10 mm × 250 mm, Cosmosil Nacalai Tesque) for the second stage separation using toluene as the eluent. The fraction marked with B1, which contained U@C_{82}(I) was collected (Figure S3a). The third stage separation for B1 was conducted on a BP column (10 mm × 250 mm, Cosmosil Nacalai Tesque) using toluene as the eluent, and pure U@C_{82}(I) was finally obtained (Figure S3b). Similarly, fraction C was re-injected into a BPM column (10 mm × 250 mm, Cosmosil Nacalai Tesque) for the second stage separation using toluene as the eluent. The fraction marked with C1, which contained U@C_{82}(II) was collected (Figure S4a). The third stage separation for C1 was conducted on a BP column (10 mm × 250 mm, Cosmosil Nacalai Tesque) using toluene as the eluent, and pure U@C_{82}(II) was finally obtained (Figure S4b). The purities of the isolated species were then reconfirmed by chromatography on a Buckyprep column (4.6 mm × 250mm, Cosmosil Nacalai Tesque) with toluene at a flow rate of 1.0 mL/min, along with the LDI-TOF mass spectrometry in a negatively charged mode (Figure 1a,1b and 1c).

Crystallographic solution of U@D_{3h}-C_{74}•Ni^{II}(OEP)•2(toluene): There are two orientations for the C_{74} cage with occupancy of 0.75 and 0.25, respectively. In the final cycles of refinement, both cages were refined with anisotropic thermal parameters. A SIMU 0.02 command was applied to smooth the displacement parameters. There is disorder involving seven U positions for the U atoms. The specific occupancies of the U positions range from 0.46 to 0.01 are as follow: U1: 0.46; U2, 0.04; U3, 0.08; U4, 0.15; U5, 0.18; U6, 0.01; U7, 0.08. All U atoms were allowed to become anisotropic. A SIMU restraint of 0.01 Å was used for U5 and U7. Besides, U1, U2 and U6 were refined with EADP due to their similar positions and high correlations. There are two benzene sites in the structure, and all of them are fully ordered.

Crystallographic solution of U@C_2(5)-C_{82}•Ni^{II}(OEP)•2(toluene). The fullerene cage is disordered with respect to a crystallographic mirror plane

that bisects the molecule, but the plane is not a symmetry element for the fullerene. Only one orientation of the cage was utilized and refined with 0.5 occupancy. All the 82 carbon atoms were refined with anisotropic thermal parameters. The displacement parameters were smoothed with SIMU 0.02. Seven disordered positions are found for the U atom in C_2(5)-C_{74}. U1 is the major site with an occupancy of 0.26, while U2, U3, U4, U5, U6, U7 have occupancies of 0.08, 0.02, 0.07, 0.03, 0.02, 0.02, respectively. The seven U positions for the uranium atom were allowed to become anisotropic in the final cycles of refinement. The refined occupancies of disordered sites for uranium atom were constrained to sum to 0.5 by the use of free variables. A SIMU restraint of 0.02 Å was used for U1 and U3. Besides, U2, U6 and U7 were refined with EADP due to their similar positions and high correlations. The structure also contains two fully ordered benzene sites.

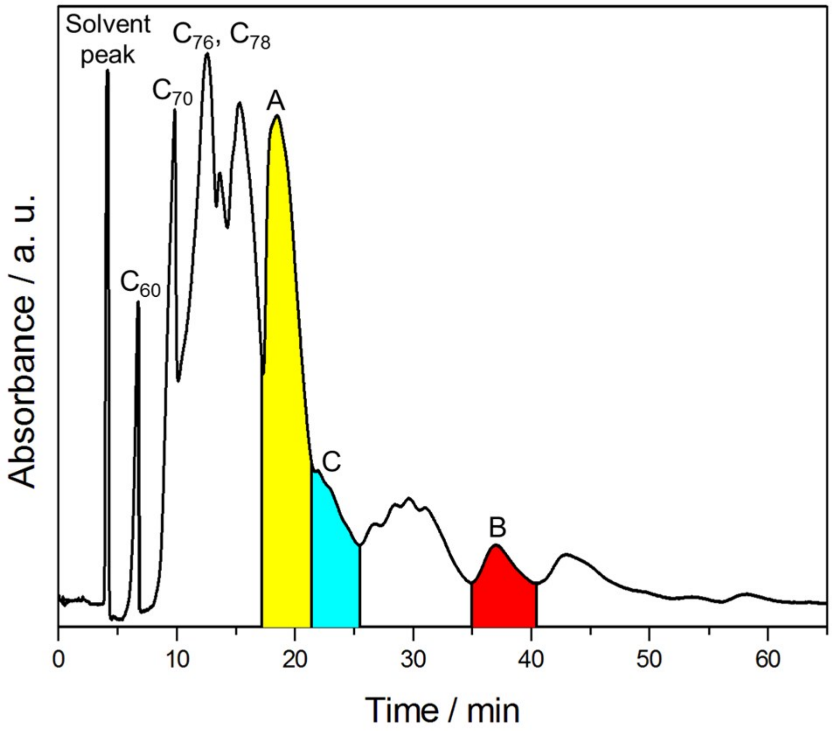

Fig. S1 The first stage HPLC chromatogram of extract. HPLC conditions: 5PYE column, Φ = 10 mm × 250 mm; eluent = toluene; flow rate = 4 mL/min; detecting wavelength = 390 nm.

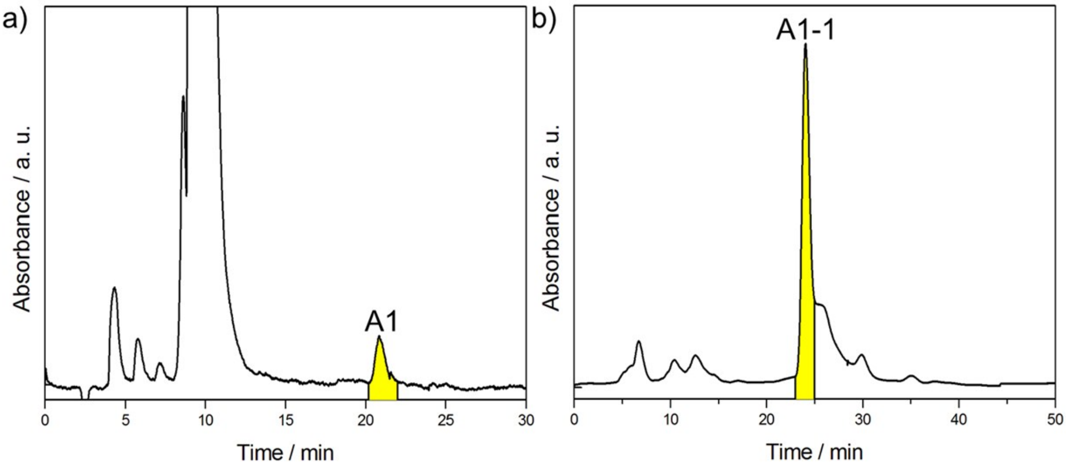

Fig. S2 (a) The second stage HPLC chromatogram of fraction A on a Buckyprep D column (*Φ* = 10 mm × 250 mm) and (b) the third stage HPLC chromatogram of fraction A1 on a Buckyprep column (*Φ* = 10 mm × 250 mm). The HPLC conditions were: eluent = toluene; flow rate = 4 mL/min; detecting wavelength = 390 nm.

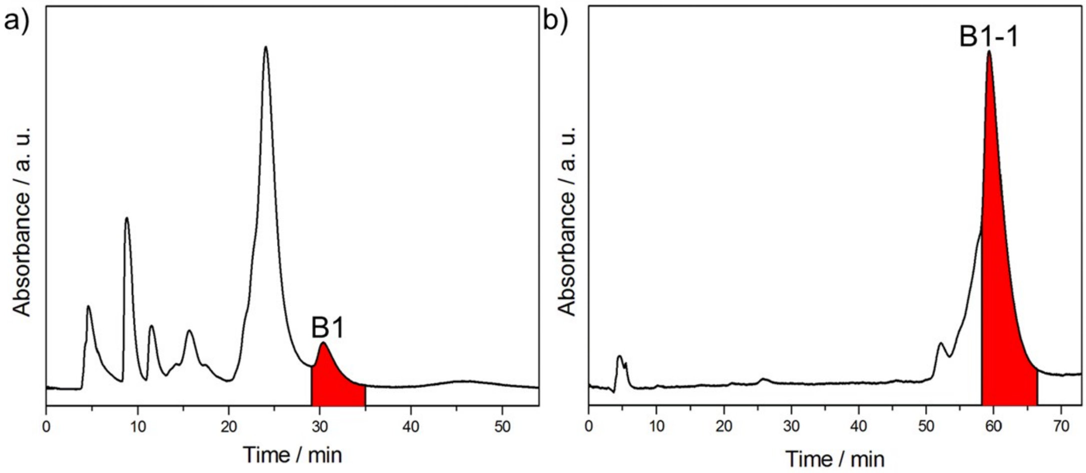

Fig. S3 (a) The second stage HPLC chromatogram of fraction B on a Buckyprep M column (*Φ* = 10 mm × 250 mm) and (b) the third stage HPLC chromatogram of fraction B1 on a Buckyprep column (*Φ* = 10 mm × 250 mm). The HPLC conditions were: eluent = toluene; flow rate = 4 mL/min; detecting wavelength = 390 nm.

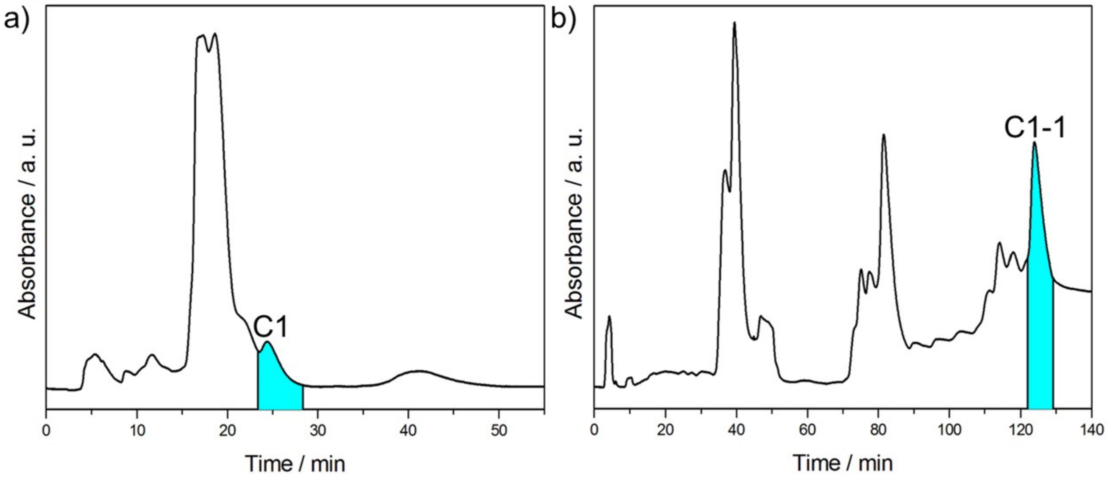

Fig. S4 (a) The second stage HPLC chromatogram of fraction C on a Buckyprep M column (Φ = 10 mm × 250 mm) and (b) the third stage HPLC chromatogram of fraction C1 on a Buckyprep column (Φ = 10 mm × 250 mm). The HPLC conditions were: eluent = toluene; flow rate = 4 mL/min; detecting wavelength = 390 nm.

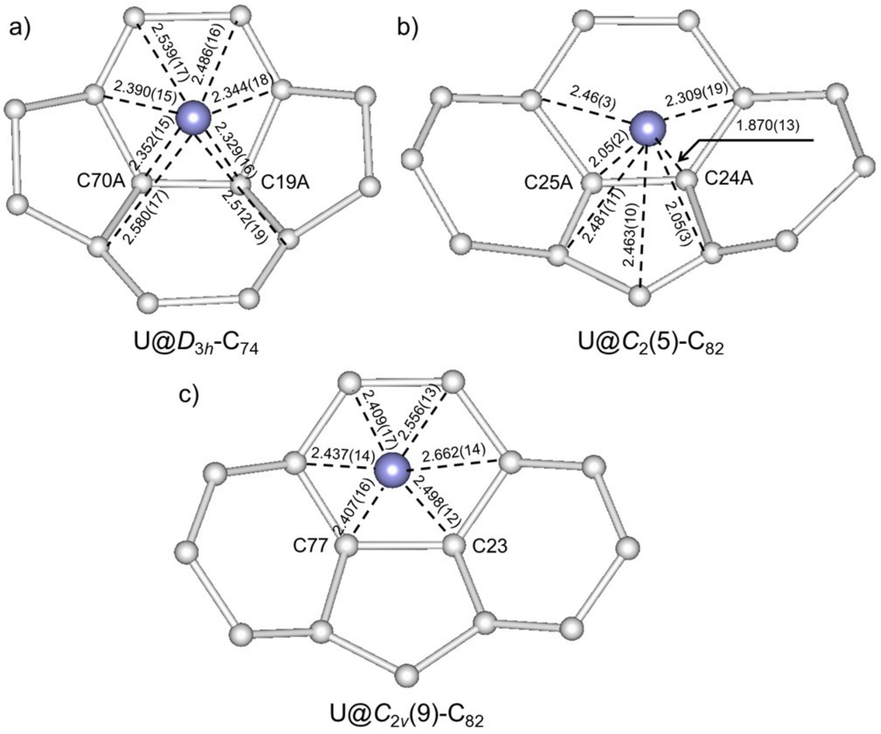

Fig. S5 Relative positions of the principal uranium site to a partial cage of (a) U@D_{3h}-C_{74}, (b) U@C_2(5)-C_{82} and (c) U@C_{2v}(9)-C_{82}.

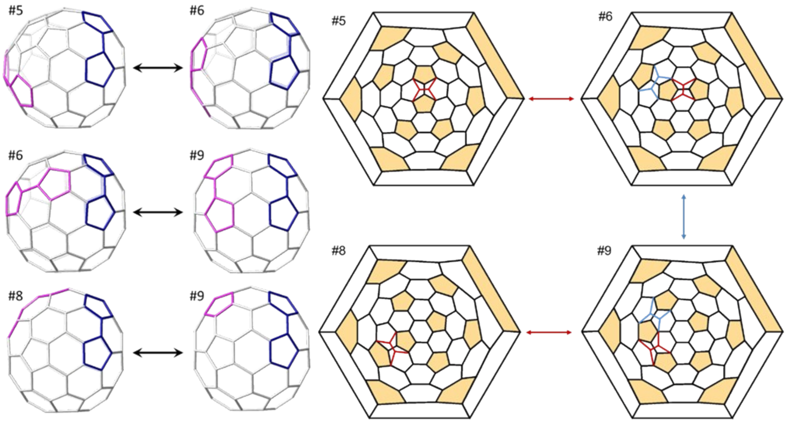

Fig. S6 Stone-Wales interconversions in isomers #5, #6, #8 and #9. Stick representation (left) where pyracylene units that take part in the interconversion are highlighted in fuchsia. Pyracylene units used as a reference are highlighted in blue. Schlegel representations (right) where Stone-Wales interconversions are schematized in red and blue lines.

Site	ΔE (kcal/mol)
1	3.3
2	3.3
3	3.3
4	0.0

Site	ΔE (kcal/mol)
1	0.0
2	13.4
3	0.0
4	24.3
5	0.0
6	0.0

Site	ΔE (kcal/mol)
1	28.3
2	0.0
3	15.1
4	8.7

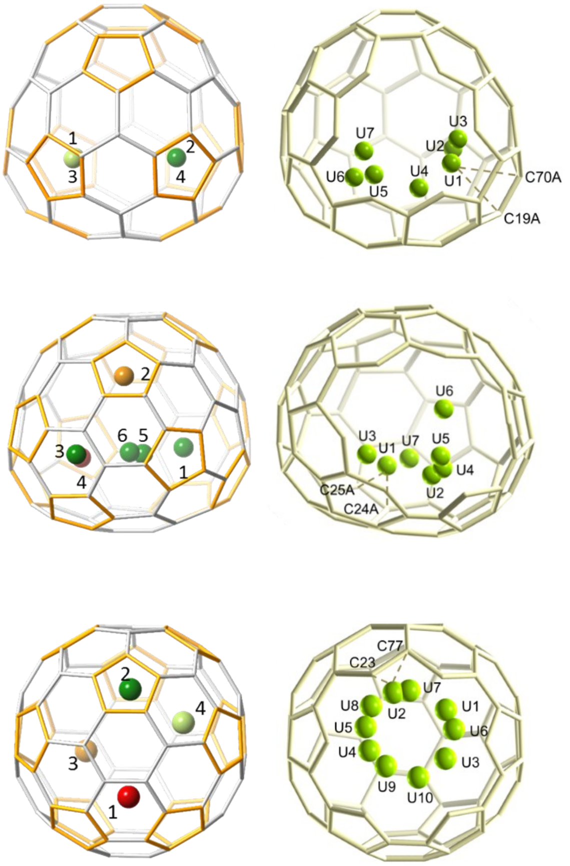

Fig. S7 Ball and stick representation for different positions of uranium in U@D_{3h}-C_{74}, U@C_2(5)-C_{82} and U@C_{2v}(9)-C_{82}, and their relative energies (kcal mol^{-1}). The U positions are colored according to the relative energies (green more favorable, red higher in

energy). On the right, perspective drawings of X-Ray structures with the disordered positions of uranium at U@D_{3h}-C_{74}, U@C_2(5)-C_{82} and U@C_{2v}(9)-C_{82}.

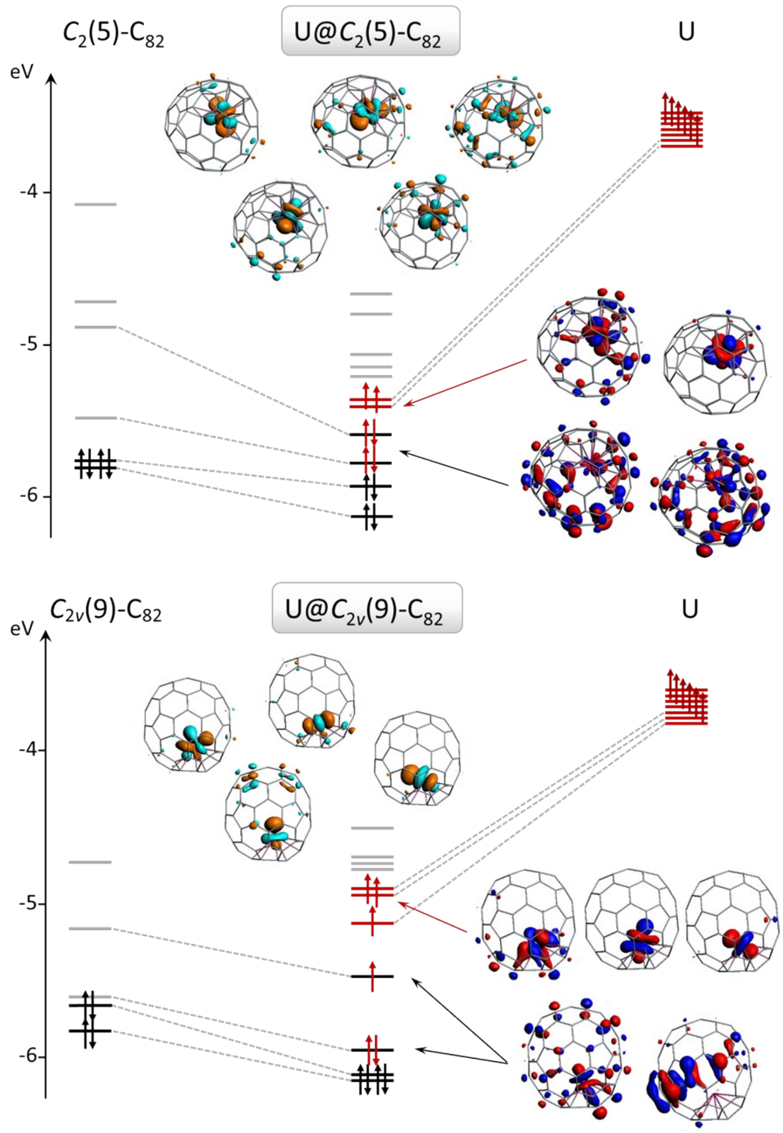

Fig. S8 Representation of the orbital interaction diagram between uranium and the C_{82} cage for U@C_2(5)-C_{82} in the triplet state (top) and U@C_{2v}(9)-C_{82} in the quintet state (bottom). Electrons from the cage and uranium are represented as black and red arrows, respectively. Same colours are used for the occupied orbitals (black for the cage and red for U) whereas empty orbitals are coloured in grey.

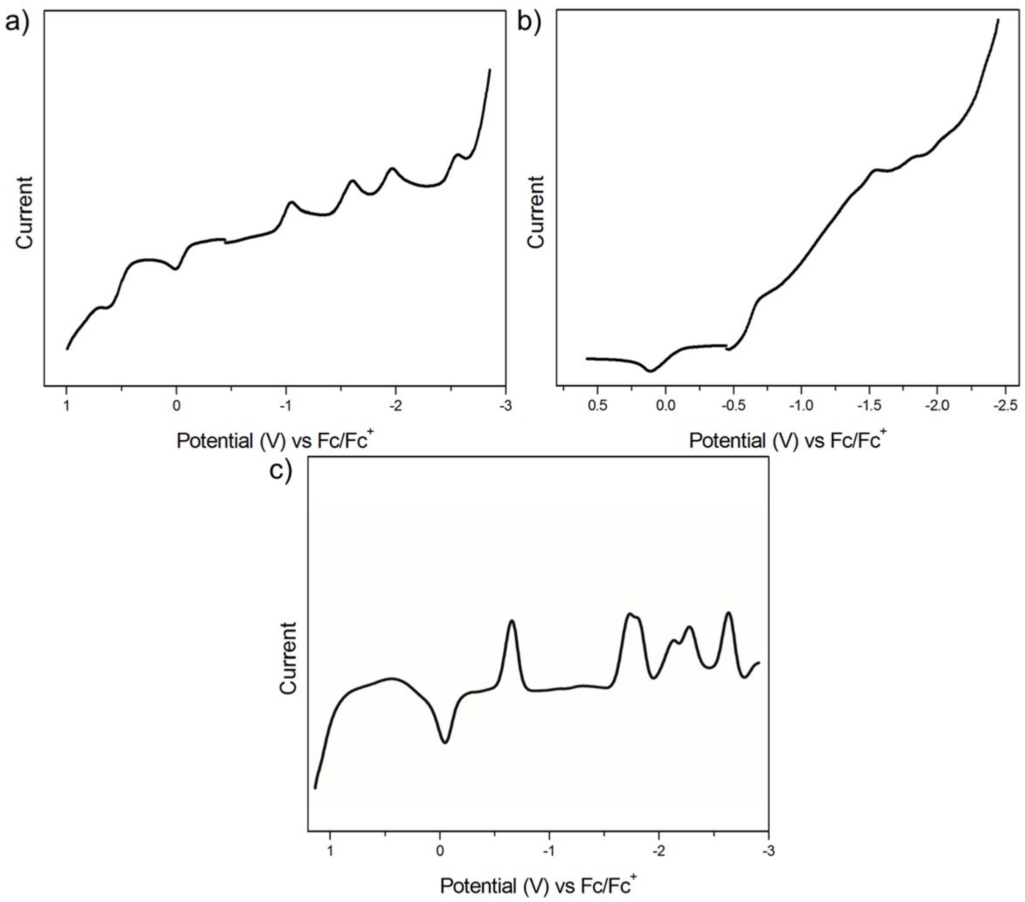

Fig. S9 Square wave voltammetry (SWV) curves of U@D_{3h}-C_{74} (a), U@C_2(5)-C_{82} (b) and U@C_{2v}(9)-C_{82} (c). Conditions: working electrode, glassy carbon disk; counter electrode, platinum wire; reference electrode, silver wire; supporting electrolyte, 0.05 M *n*-Bu_4NPF_6 in 1,2-dichlorobenzene. Pulse amplitude, 50mV. Pulse width, 50ms. Pulse period, 500ms.

Table S1. Relative energies (in kcal mol^{-1}) for U@C_{82}. Values using scalar relativistic corrections (SR) and spin-orbit corrections (SO).

Isomer	U@C_{82} SR	U@C_{82} SO
C_2(5)-C_{82}	1.9	3.4
C_s(6)-C_{82}	4.7	5.3
C_{3v}(8)-C_{82}	2.2	3.5
C_{2v}(9)-C_{82}	0.0	0.0

Table S2. Experimental and Computed First Anodic and Cathodic Potentials for Uranofullerenes.a, b)

Species	$^{ox}E_1$ [V]	$^{red}E_1$ [V]	ΔE_{gap} [V]^c
U@D_{3h}-C_{74}	0.01	-1.05	1.06
	[-0.14]	[-0.90]	[0.76]
U@C_2(5)-C_{82}	0.11	-0.67	0.78
	[0.15]	[-0.78]	[0.93]
U@C_{2v}(9)-C_{82}	0.10	-0.43	0.53
	[0.07]	[-0.46]	[0.53]

a) Computed values in square brackets; b) potential and gaps in V; c) electrochemical gaps.

Table S3. Estimated oxidation states for U-EMFs.

	**U@D_{3h}-C_{74}**	**U@C_2(5)-C_{82}**	**U@C_{2v}(9)-C_{82} (triplet)**	**U@C_{2v}(9)-C_{82} (quintet)**
Neutral	U^{4+}@D_{3h}-C_{74}^{4-}	U^{4+}@C_2(5)-C_{82}^{4-}	$U^{3.5+}$@C_{2v}(9)-$C_{82}^{3.5-}$	U^{3+}@C_{2v}(9)-C_{82}^{3-}
Oxidized	U^{5+}@D_{3h}-C_{74}^{4-}	U^{4+}@C_2(5)-C_{82}^{3-}	U^{4+}@C_{2v}(9)-C_{82}^{3-}	U^{4+}@C_{2v}(9)-C_{82}^{3-}
Reduced	U^{3+}@D_{3h}-C_{74}^{4-}	U^{3+}@C_2(5)-C_{82}^{4-}	U^{3+}@C_{2v}(9)-C_{82}^{4-}	U^{3+}@C_{2v}(9)-C_{82}^{4-}

75

U@C_{74} IPR1 (Binding Energy= -651.34 eV)

C	1.959332	1.753622	-3.138082
C	0.863829	2.692080	-3.073944
C	-0.320720	2.014485	-3.547580
C	0.031841	0.654807	-3.891646
C	1.456122	0.491913	-3.633180
C	2.943826	1.760908	-2.147469
C	2.844496	2.683285	-1.032872
C	1.771301	3.568561	-0.954408
C	0.773004	3.589004	-1.997068
C	-0.501386	3.828612	-1.362602
C	-1.648581	3.198562	-1.841062
C	-1.558019	2.277639	-2.956713

C	-2.498950	1.213395	-2.732468
C	-2.184990	-0.118123	-3.096344
C	-0.878993	-0.406314	-3.690497
C	-0.308765	-1.721630	-3.476713
C	1.095068	-1.877270	-3.218246
C	1.983088	-0.733724	-3.169525
C	3.040826	-0.717894	-2.155570
C	3.494602	0.528066	-1.656473
C	3.770766	0.709536	-0.240124
C	3.334099	2.020575	0.149176
C	2.742782	2.251103	1.428522
C	1.590658	3.160615	1.509345
C	1.114734	3.780029	0.317595
C	-0.292693	3.941305	0.064892
C	-1.272147	3.488898	0.995464
C	-2.495719	2.850917	0.487843
C	-2.646715	2.705672	-0.925109
C	-3.198175	1.507877	-1.491299
C	-3.669524	0.464216	-0.655427
C	-3.492630	-0.877805	-1.112328
C	-2.797730	-1.171333	-2.330874
C	-2.248234	-2.497419	-2.217940
C	-1.003284	-2.827850	-2.852432
C	-0.030536	-3.707803	-2.195270
C	1.268148	-3.070770	-2.424964
C	2.263593	-2.994950	-1.399366
C	3.117989	-1.847073	-1.270830
C	3.404978	-1.671616	0.124365
C	3.690723	-0.380322	0.664929
C	3.277387	-0.117402	1.996007
C	2.840324	1.194246	2.383365

C	1.857293	1.032876	3.424520
C	0.779532	1.910055	3.520331
C	0.622657	2.957752	2.535651
C	-0.785079	3.119013	2.282758
C	-1.495214	2.169688	3.111043
C	-2.639343	1.547591	2.618212
C	-3.144204	1.879515	1.309393
C	-3.696971	0.681492	0.744552
C	-3.477180	-0.416923	1.673696
C	-3.146402	-1.714558	1.210968
C	-3.274968	-1.962054	-0.198640
C	-2.541605	-2.985389	-0.897341
C	-1.588542	-3.805210	-0.202152
C	-0.331475	-4.206095	-0.836119
C	0.684526	-4.039461	0.201371
C	1.972743	-3.482853	-0.081817
C	2.648318	-2.640916	0.866739
C	2.092501	-2.313953	2.148861
C	2.518567	-1.103022	2.750508
C	1.660600	-0.386200	3.651682
C	0.387573	-0.874699	3.958000
C	-0.724323	0.039686	4.086442
C	-0.529609	1.411761	3.874647
C	-1.895403	-0.613120	3.546100
C	-2.847376	0.129844	2.842890
C	-1.510852	-1.917776	3.063564
C	-2.127155	-2.485653	1.926896
C	-1.339708	-3.440344	1.173946
C	0.066466	-3.592947	1.425266
C	0.742833	-2.810029	2.438273
C	-0.082997	-2.081235	3.321095

U	-0.146637	-1.836182	-0.747743

83

U@C_{82} IPR5 (Binding Energy= -722.18 eV)

C	-4.384164	-0.249292	0.951382
C	-4.085588	-1.616183	0.564687
C	-3.902329	-1.656039	-0.853330
C	-4.166585	-0.322046	-1.355536
C	-4.423470	0.555126	-0.241434
C	-3.850193	0.266152	2.129269
C	-3.005174	-0.560484	2.957291
C	-2.764369	-1.888925	2.608955
C	-3.310476	-2.427314	1.385748
C	-2.322552	-3.308019	0.801234
C	-2.051979	-3.319886	-0.596284
C	-2.900700	-2.480673	-1.448691
C	-2.384498	-2.050233	-2.701994
C	-2.703671	-0.760881	-3.247153
C	-3.537470	0.150881	-2.536779
C	-3.238852	1.541107	-2.608903
C	-3.456643	2.399998	-1.488995
C	-3.957261	1.901265	-0.243728
C	-3.402675	2.451517	0.997176
C	-3.328506	1.611304	2.146732
C	-2.169345	1.608806	2.997810
C	-1.924606	0.253302	3.441437
C	-0.606200	-0.247697	3.576950
C	-0.368643	-1.663849	3.266770
C	-1.457104	-2.447589	2.807458
C	-1.258637	-3.429614	1.761780
C	0.008060	-3.768057	1.342472

C	0.280501	-3.976949	-0.052869
C	-0.692546	-3.669700	-1.042381
C	-0.199584	-3.186100	-2.336360
C	-1.065116	-2.396508	-3.134754
C	-0.566809	-1.320110	-3.958223
C	-1.572131	-0.299599	-4.000865
C	-1.212053	1.082137	-3.998538
C	-2.063420	2.003184	-3.328522
C	-1.534675	3.138440	-2.637582
C	-2.422514	3.401714	-1.529572
C	-1.924147	3.954510	-0.365815
C	-2.416734	3.481408	0.916136
C	-1.322504	3.538792	1.827513
C	-1.110588	2.524026	2.810994
C	0.239269	2.091620	3.142003
C	0.487385	0.717807	3.509871
C	1.834397	0.225806	3.327998
C	2.025991	-1.128046	2.910875
C	0.968499	-2.084411	2.861926
C	1.149414	-3.153675	1.959162
C	2.219953	-3.138182	0.978426
C	1.679800	-3.675005	-0.242186
C	2.130088	-3.200145	-1.466119
C	1.204723	-2.992074	-2.534843
C	1.708111	-1.888718	-3.321742
C	0.819558	-1.002269	-3.996174
C	1.185517	0.380145	-4.091791
C	0.177541	1.409911	-4.051523
C	0.703151	2.523808	-3.320132
C	-0.127152	3.356044	-2.522347
C	0.418094	3.900850	-1.279672

C	-0.522266	4.265467	-0.257648
C	-0.193992	4.092479	1.116130
C	1.087172	3.737955	1.489358
C	1.345149	2.833252	2.577197
C	2.694173	2.348553	2.430859
C	2.968232	1.005993	2.860409
C	3.796937	0.132178	2.035758
C	3.174813	-1.175068	2.046742
C	3.198634	-2.118603	0.955547
C	3.695910	-1.636089	-0.331554
C	3.158817	-2.188244	-1.519723
C	2.901415	-1.393325	-2.696515
C	3.189975	-0.000902	-2.696776
C	2.349620	0.872914	-3.430141
C	2.048528	2.200645	-2.926582
C	2.596070	2.690772	-1.727447
C	1.776285	3.560654	-0.882344
C	2.130475	3.589276	0.515584
C	3.202455	2.831611	1.140849
C	3.990657	1.959342	0.311101
C	4.306065	0.610143	0.759618
C	4.147918	-0.265916	-0.385512
C	3.824379	0.534162	-1.534604
C	3.618757	1.886202	-1.088667
U	1.877147	0.818535	0.694017

83

U@C_{82} IPR9 (Binding Energy= -722.26 eV)

C	1.266218	-0.908257	-4.012013
C	-1.162985	-0.887530	-4.016930
C	0.046886	-1.645514	-3.962261

C	1.281182	0.521579	-3.932283
C	-1.152418	0.541159	-3.938133
C	0.069691	1.257836	-3.849359
C	2.445432	-1.375837	-3.322945
C	-2.348822	-1.336023	-3.327550
C	2.452015	0.928631	-3.176390
C	-2.313264	0.976749	-3.182058
C	0.035722	-2.934427	-3.256745
C	0.075476	2.455824	-3.054589
C	3.146049	-0.257535	-2.752328
C	-3.023727	-0.205822	-2.754332
C	2.441576	-2.585906	-2.659681
C	-2.364648	-2.546985	-2.659396
C	1.238622	-3.398541	-2.646611
C	-1.173621	-3.379925	-2.646446
C	1.231510	2.878722	-2.339965
C	-1.076617	2.946478	-2.363943
C	2.426864	2.078646	-2.356360
C	-2.265710	2.134231	-2.373936
C	3.755171	-0.350736	-1.463747
C	-3.616451	-0.292770	-1.463697
C	3.129716	-2.732608	-1.405052
C	-3.053019	-2.684645	-1.407329
C	1.231078	-4.135720	-1.422080
C	-1.177168	-4.118619	-1.423261
C	0.810035	3.689725	-1.222982
C	-0.657690	3.782447	-1.240237
C	3.187314	2.054706	-1.160750
C	-2.998310	2.125809	-1.154294
C	3.715790	-1.617902	-0.733663
C	-3.613511	-1.558133	-0.733246

C	2.425958	-3.758615	-0.687389
C	-2.364532	-3.723017	-0.687123
C	0.025280	-4.444707	-0.735717
C	3.840628	0.862481	-0.718888
C	-3.637663	0.921254	-0.711969
C	1.569428	3.607682	0.000000
C	-1.437200	3.810731	0.000000
C	2.743732	2.793229	0.000000
C	-2.589550	2.910343	0.000000
C	3.840628	0.862481	0.718888
C	-3.637663	0.921254	0.711969
C	0.025280	-4.444707	0.735717
C	2.425958	-3.758615	0.687389
C	-2.364532	-3.723017	0.687123
C	3.715790	-1.617902	0.733663
C	-3.613511	-1.558133	0.733246
C	3.187314	2.054706	1.160750
C	-2.998310	2.125809	1.154294
C	0.810035	3.689725	1.222982
C	-0.657690	3.782447	1.240237
C	1.231078	-4.135720	1.422080
C	-1.177168	-4.118619	1.423261
C	3.129716	-2.732608	1.405052
C	-3.053019	-2.684645	1.407329
C	3.755171	-0.350736	1.463747
C	-3.616451	-0.292770	1.463697
C	2.426864	2.078646	2.356360
C	-2.265710	2.134231	2.373936
C	1.231510	2.878722	2.339965
C	-1.076617	2.946478	2.363943
C	1.238622	-3.398541	2.646611

C	-1.173621	-3.379925	2.646446
C	2.441576	-2.585906	2.659681
C	-2.364648	-2.546985	2.659396
C	3.146049	-0.257535	2.752328
C	-3.023727	-0.205822	2.754332
C	0.075476	2.455824	3.054589
C	0.035722	-2.934427	3.256745
C	2.452015	0.928631	3.176390
C	-2.313264	0.976749	3.182058
C	2.445432	-1.375837	3.322945
C	-2.348822	-1.336023	3.327550
C	0.069691	1.257836	3.849359
C	1.281182	0.521579	3.932283
C	-1.152418	0.541159	3.938133
C	0.046886	-1.645514	3.962261
C	1.266218	-0.908257	4.012013
C	-1.162985	-0.887530	4.016930
U	-0.327954	1.727405	0.000000
